# Possibilities of the Direct Chemical Transformation of Kukersite Kerogen: A Critical Review

**DOI:** 10.1021/acsomega.5c04675

**Published:** 2025-09-02

**Authors:** Margus Lopp, Kristiina Kaldas

**Affiliations:** Department of Chemistry and Biotechnology, Laboratory of Industrial Chemistry, 54561 Tallinn University of Technology, Tallinn 19086, Estonia

## Abstract

The Lille-Blokker model of the chemical structure of kukersite oil shale provides a good basis for developing methods for its direct conversion into new materials and basic organic chemicals. The model of its structure may be considered a complex organic “macromolecule”. Existing information on the chemical treatment of kukersite kerogen by hydrogenation, halogenation, alkylation, sulfonation, chloromethylation, oxidation, etc., is critically reviewed, and different options for bulk kukersite conversion are discussed. So far, the direct conversion approach has only been applied to the oxidation of kukersite to obtain dicarboxylic acids. In this paper, a chemical reactivity map of kukersite kerogen is presented, highlighting possible pathways for its transformation. This study aims to demonstrate that, within the context of expanding research on unconventional resources, kerogen can serve as a focal point in materials-oriented science.

## Graphical abstract

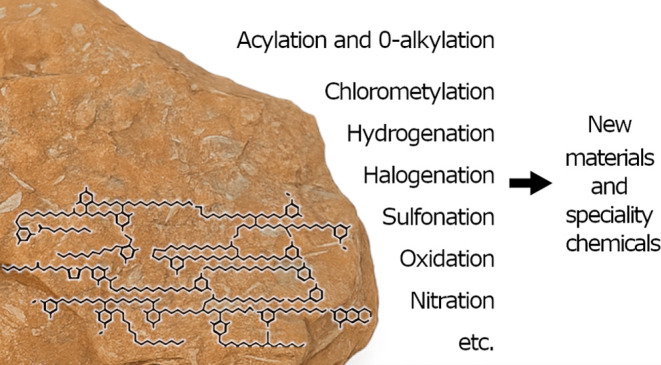

## 1 Introduction

Oil shale is a sedimentary rock rich in organic matter (OM) called kerogen, the content of which may have a lower limit of 10% and an upper limit of 60%.^1^ Oil shale is one of the most abundant fossil fuel reserves on Earth, occurring in more than 40 countries.^2–4^ The largest oil shale deposits are in the United States, Russia, Brazil, China, Jordan, Australia, and Estonia.^4–12^ The main characteristics of different oil shales are presented in Table 1.

**Table 1:** Main Characteristics of Different Oil Shales and Its Kerogens^a^ ^3–5,7–9^

country	deposit	age	origin	van Krevelen type	OM wt %	C in ker, wt %	H/C in kerogen	O/C in kerogen	oil yield %
USA	Green River	Eocene	lacustrine	I	16–21	80–81	1.5–1.6	0.05–0.07	10–11
Jordan	Lajjun	Cretaceous	marine	I/II	22–28	68–69	1.3–1.4	0.06–0.1	8–12
Estonia	Estonia	Ordovician	marine	I/II	30–45	76–77	1.4–1.5	0.13–0.16	20–22
China	Fushun	Tertiary	terrestrial	I	19–21	80	1.5	0.07	7–8
Australia	Stuart	Tertiary	lacustrine	I	27	84	1.6	0.03	9–14
Brazil	Irati	Premian	marine	II	16–30	68	1.8	0.18	7–8

a Table reproduced from the doctoral dissertation of Kaldas^13^ with the authors permission.

The present article is devoted to kukersite oil shale, found mainly in Estonia, which, according to the van Krevelen diagram, is classified between Type I and Type II oil shale. Kukersite was formed in the Ordovician period over 450 million years ago and contains 20–70% organic matter. It has mainly been used for energy and oil production^14–16^ by thermal decomposition; an industrial method for producing oil from kukersite was developed already in the 1920s^17,18^ and in 2023, more than 1.2 million tons of oil were produced from kukersite.^19^ Oil production methods from other oil shales have also been developed and comprehensive overviews of these processes can be found elsewhere.^20–22^

At the beginning of the 20th century, Zalessky^23,24^ and Fokin^25^ suggested that kukersite kerogen was formed mainly from *Gloeocapsomorpha Prisca* microorganisms, and this assumption was confirmed more than 70 years later by Forster et al.^26^ In spite of extensive work, the chemical structure of kukersite remained unknown for more than a hundred years.^27–38^

In the early 1970s, Ülo Lille noticed^39^ that 1,3-benzenediol structural units serve as markers of a biological origin from extant algae.^40–49^ He proposed that the main structural elements of native kerogen are derivatives of 1,3-benzenetriols of microbial origin, connected to each other by aliphatic carbon chains, and he offered a general model of the kukersite kerogen structure.^50,51^ At the same time, Blokker et al. performed an oxidation of kukersite kerogen with RuO_4_ and found that kukersite kerogen consists of 1,3-benzenetriol rings linked primarily by aliphatic carbon chains.^52^

The models of Lille and Blokker are fundamentally very similar. Therefore, we have generalized these models and presented a unified, simplified Lille-Blokker structural model for kukersite kerogen (Scheme 1).^53^

**Scheme 1:**
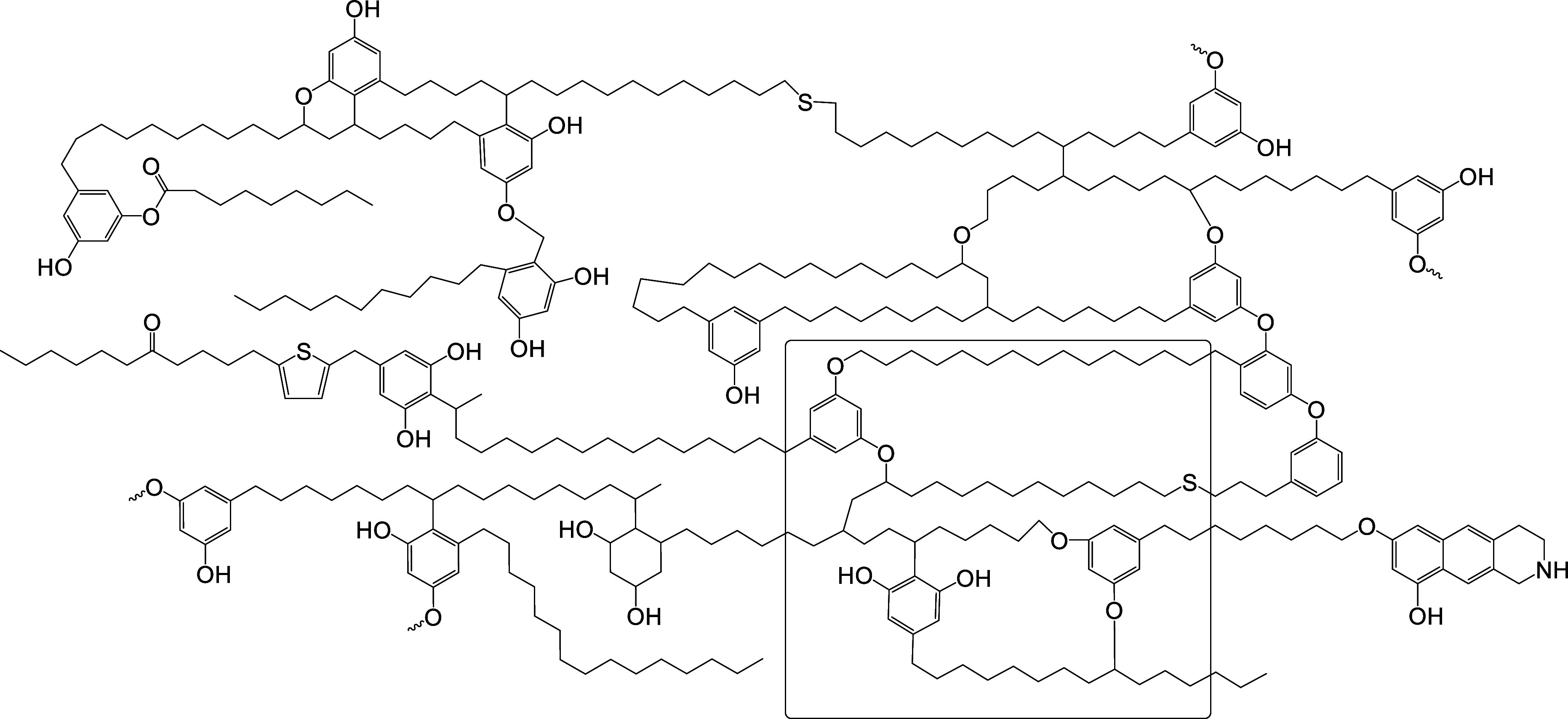
Simplified Lille-Blokker Structural Model of Kukersite Kerogen as a Hypothetical Kerogen “Molecule” With the Molecular Weight 5709,85D; A Mean Fragment of It is Selected in the Rectangle and Used to Demonstrate Possible Chemical Reactions

Of course, structural models of other kerogens have also been proposed and discussed in the literature.^54–56^ For example, kukersite, Beipiao, and Fushun oil shales are quite similar in their elemental composition, but they differ significantly in their aromatic structures: kukersite contains exclusively resorcinolic-type aromatic units, whereas Beipiao and Fushun oil shales contain other aromatic fragments and completely lack resorcinolic units. Therefore, each kerogen requires a different approach to determine the possible chemical transformations applicable and in this review, exclusive focus is placed on kukersite.

The Lille-Blokker structural model of kukersite may serve as a basis for its chemical transformation. Surprisingly, there are only a few publications that analytically examine the chemical reactions of kukersite. To date, almost no studies have addressed the use of oil shale as a readily available and inexpensive raw material for the preparative production of basic organic chemicals. The only notable exception is the well-studied oxidation of kukersite to obtain dicarboxylic acids^57,58^ (see Section 2.7). This stands in striking contrast to lignin, another major natural resource derived from wood processing, for which numerous structure-based studies on its transformation have been conducted. A wide range of chemical reactions of lignin have been studied in order to develop new materials, building blocks and chemicals^59,60^ including the following: fragmentation of the lignin backbone,^61^ oxidation,^62^ esterification,^63^ sulfonation,^64^ halogenation,^65^ nitration,^66^ alkylation/dealkylation,^67–69^ hydroxyalkylation,^70,71^ amination,^72–74^ reactions with isocyanates^75,76^ etc.

In this paper, we examine the possibilities for different chemical pathways to transform kukersite kerogen into valuable chemicals and materials, thereby providing a chemical reactivity map and promoting corresponding preparative studies.

## 2 Chemical Reactivity and Reactions of the Kukersite “Molecule”

### 2.1 Reactive Sites in the Kukersite Structural Unit

First, we will look at the kukersite structural unit as a complex organic molecule. The Lille - Blokker kukersite “molecule” has only a few specific functional groups. The most distinguished of them are the alkyl resorcinol units, forming the backbone of the structure. Based on literature data, it has been established that ∼20% of the carbons are aromatic, and ∼75% of kerogen carbons correspond to aliphatic carbons with the average chain length of ∼7.5 methylene groups. According to the model, the aromatic groups predominately contain two oxygen atoms in the *m*-position, making them resorcinolic. The oxygens appear as free phenolic hydroxyl groups and as phenolic ether groups. The exact ratio of free to substituted aromatic hydroxyl (OH) groups has not been established, but it is estimated to be less than 50%.^51,52^ The oxygen-bound aliphatic carbons represent 4–5%, being mostly ethers.^77^ Based on available data, a hypothetical kukersite “molecule” is estimated to contain around 16 resorcinol units. The hydrocarbon part mainly consists of unbranched, branched or cyclic aliphatic chains attached to the resorcinol carbon or oxygen. A simplified fragment of the “molecule” represents the main structural units of kukersite kerogen. By analyzing the possible chemical reactions of kukersite based on the fragment shown in Scheme 1, several reactive sites can be identified, as presented in Scheme 2.

**Scheme 2:**
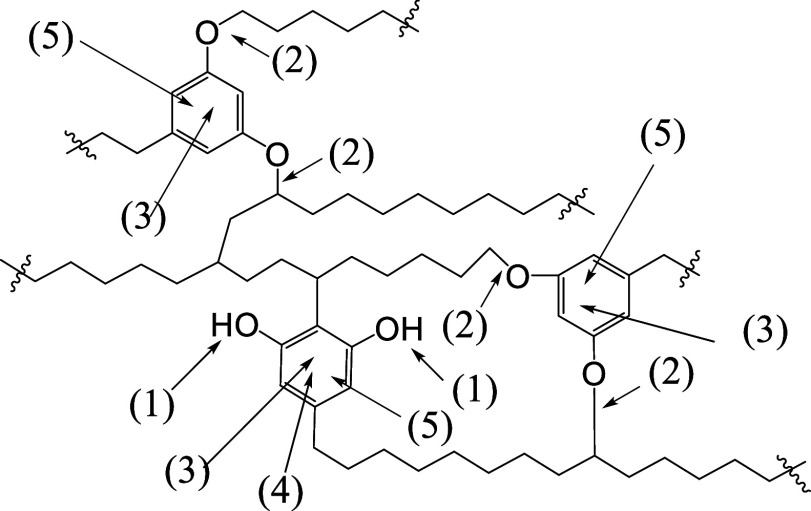
Reactive Sites of the Aromatic Part of Kukersite

Scheme 2 implies a variety of chemical reactions that can be applied to modify the native structure of kukersite kerogen, including: (1) alkylation or acylation of free phenolic OH groups, (2) dealkylation of the phenol ether group, (3) substitution reactions in the aromatic ring, such as halogenation, alkylation, hydroxymethylation and chloromethylation, nitration, sulfonation etc., (4) destructive oxidation of the resorcinol unit bearing free OH groups, and (5) oxidation and reduction of the aromatic ring etc.

In addition, the hydrocarbon component is also susceptible to chemical modification. In Scheme 3 possible chemical transformation reactions on the hydrocarbon part of kukersite are presented. First, the reactions with active benzylic hydrogens may be considered. The benzylic sites of the “molecule” can be (1) halogenated, (2) oxidized, (3) reduced, or (4) radically substituted. The hydrocarbon branched carbons may additionally rearrange, disproportionate or undergo other structural changes during transformations, showing the complexity and diversity of possible reaction pathways.

**Scheme 3:**
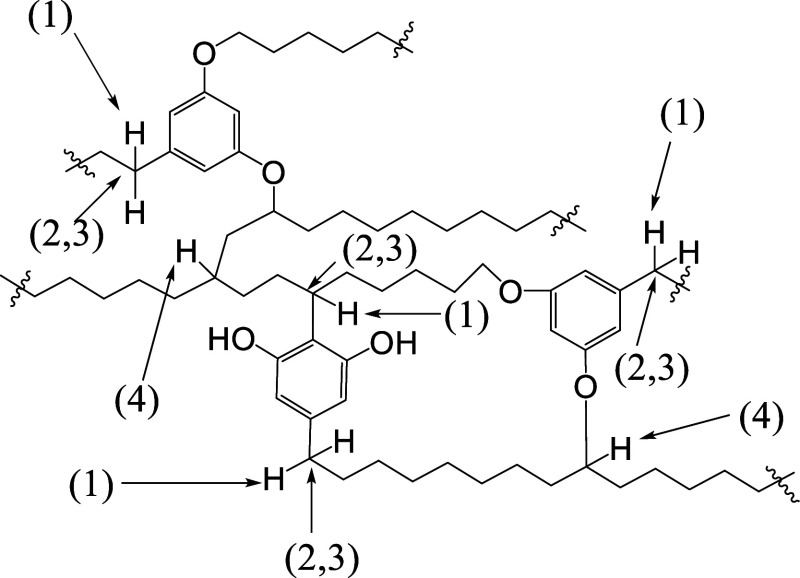
Reactive Sites in the Aliphatic Part of the “Molecule” Fragment of Kukersite

Many of these reactions may be performed selectively, providing an opportunity for the directed transformation of kukersite kerogen. In this paper, the possibilities of these chemical transformations are examined by using the existing literature data and are evaluated in relation to the kukersite “molecule”.

### 2.2 Hydrogenation

Hydrogenation of kerogen transforms unsaturated structural units into saturated ones. In addition, dehydroxylation of alcohol units also occurs. The hydrogenation of kukersite was studied by Kogerman and Kopwillem.^78^ They used concentrated kukersite (47.4% of the kerogen content) and performed hydrogenation in an autoclave under pressure of hydrogen in the presence of Fe_2_O_3_ at temperatures up to 420 °C for 4 h. In the course of the reaction, they observed a pressure decrease of 24 kg/cm^3^. If we calculate the amount of hydrogen available for the hydrogenation of aromatic rings using the hypothetical “molecule”, it appears that every resorcinol unit adds roughly two or three hydrogen atoms: 30% of resorcinol units add three hydrogens, 70% of resorcinol units add two hydrogens, and 50% resorcinols contain free OH groups that, through water elimination reactions, incorporate an additional two hydrogens. As a result, the theoretical hydrogen consumption of the “molecule” should be 1.929 g-atoms. From the reported hydrogenation experiment, 1.925 g-atoms of hydrogen were added to the kerogen “molecule” (Scheme 4). This is in good accordance with the model and suggests that it reliably reflects the actual structure of kukersite kerogen.

**Scheme 4:**
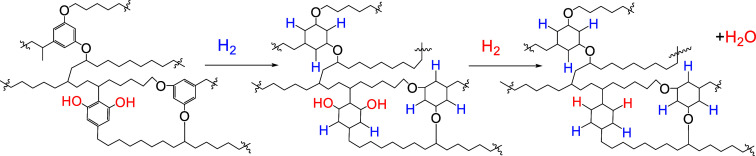
Hydrogenation Pathways in the Fragment of Kukersite Hypthetic “Molecule”

There have also been some studies on the hydrogenation of other types of shale.^79,80^ It can be assumed that preliminary hydrogenation simplifies the molecular structure of kerogen and, when followed by a thermal process, may facilitate the direct production of useful hydrocarbons from oil shale.

### 2.3 Halogenation

Halogenation of kerogen converts double bonds and its structural units containing benzylic hydrogens into chlorinated structures. The halogenation of kukersite was already studied by Paul Kogerman in the course of his investigations into the structure of kerogen. He carried out a halogenation of kukersite concentrate with chlorine gas and a bromination with Br_2_.^81^ He found that 0.25 g of chlorine reacts with 1 g of kukersite kerogen in a substitution reaction, together with the formation of hydrogen chloride gas. From the Lille-Blokker model, we may suggest that chlorine substitutes benzylic and secondary hydrogens from the branched carbons, resulting in chlorinated kerogen (Scheme 5).

**Scheme 5:**
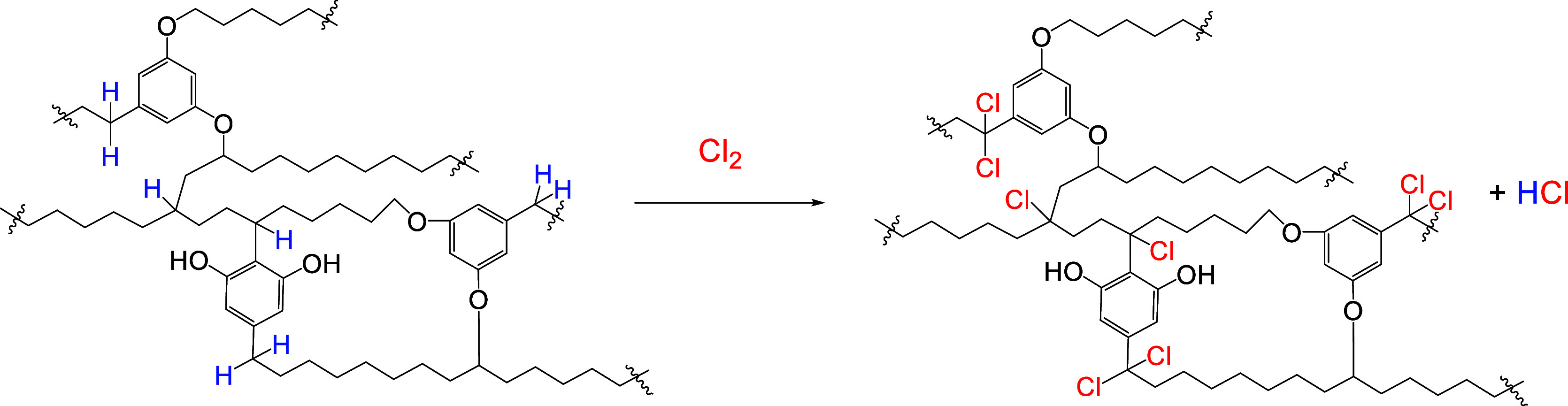
Substitution of Chlorine in the Fragment of the Hypothetic “Molecule” of Kerogen

Kogerman also determined that the increase in kerogen weight was 41.7%, i.e. 67 atoms of chlorine were added to the hypothetic “molecule”. However, the estimated number of benzylic and secondary hydrogens in the Lille-Blokker “molecule” that can participate in chlorine substitution reaction is 54. That is less than the added chlorine atoms. As the number of resorcinol units is relatively well established, the result may indicate that the proportion of branching in the current Lille-Blokker model has been underestimated.

Proskurjakov et al. also chlorinated kukersite with Cl_2_ and obtained a product containing ≥30% of Cl.^82,83^ This indicates that more than 48 chlorine atoms were added to the “molecule,” which is also in good agreement with previous results. He also found that the amount of chlorine consumed increased when a FeCl_3_ catalyst was added. They suggested that this was caused by a Cl addition to double bonds, but no analytical evidence was presented. Based on the Lille-Blokker model, it is highly likely that in the presence of a Lewis acid the additional consumption of chlorine was caused by aromatic electrophilic substitution reactions to the resorcinol nuclei.

Chlorination also increases the solubility of kerogen in organic solvents so that 15% of the kerogen is dissolved.^82^ The soluble part of kerogen can be used in different homogeneous chemical reactions to transform kukersite kerogen to valuable products.

Therefore, chlorination offers new opportunities for the transformation of kukersite kerogen. The chlorine atoms in chlorinated kerogen can be further hydrolyzed to form alcohols, ketones, and carboxylic acids, thereby affording partially oxidized kerogen units (Scheme 6).

**Scheme 6:**
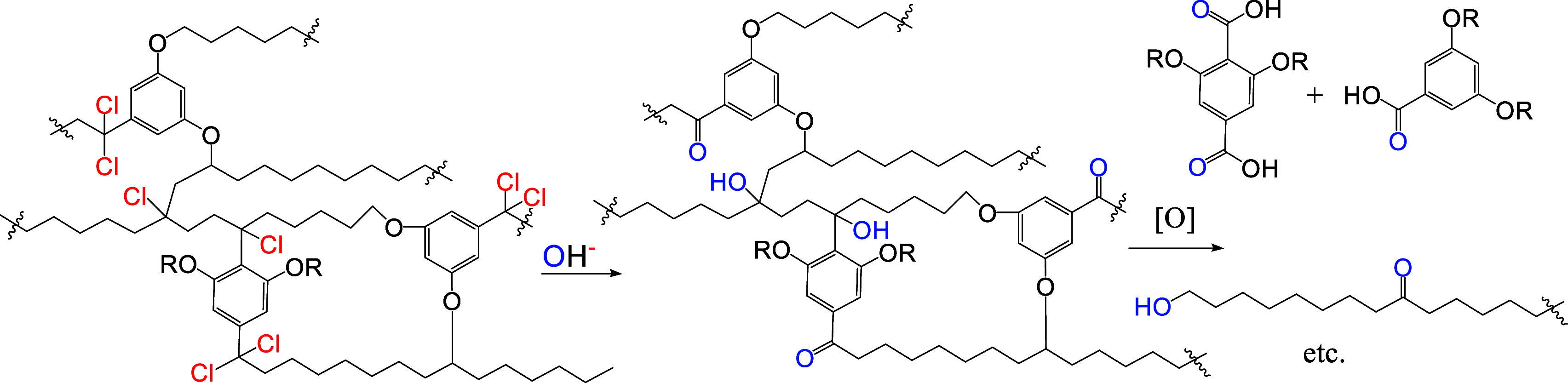
Hydrolysis and Oxidation of Chlorinated “Molecule” Fragment

This also opens the possibility of protecting the phenolic OH groups in the hydrolyzed intermediates and subsequently oxidizing them under mild conditions. Thus, the chlorination may act as a “pretreatment” step before the main destructive reactions. In this way, the chlorination/hydrolysis/protection/oxidation sequence can be used to produce chemicals from kerogen.

### 2.4 *O*-Alkylation and Acylation

*O*-Alkylation transforms the hydroxyl moieties of kerogen into ethers, and acylation converts them into esters. Resorcinolic hydroxyl groups in kukersite kerogen are the most characteristic and promising sites for such derivatization. These OH groups are either free or alkylated with long hydrocarbon chains. However, the ratio of free/alkylated hydroxyls was not exactly defined by Lille and Blokker. The free resorcinolic hydroxyl groups in kerogen may be alkylated to afford resorcinol ethers, or acylated to give aromatic esters.

The first, but unsuccessful, attempt to alkylate kukersite kerogen with dimethyl sulfate was mentioned by Aarna and Lippmaa.^37^ When we recently attempted to alkylate native finely ground dry kukersite kerogen (dried for 24 h at 220 °C) with dimethyl carbonate as a nontoxic “green” alkylating agent,^84^ we observed an almost quantitative disappearance of the OH signal by IR and the appearance of an OCH_3_ carbon peak in a solid-state NMR spectrum.^85^ According to our estimation, the percentage of free OH groups is under 50% (Scheme 7).

**Scheme 7:**
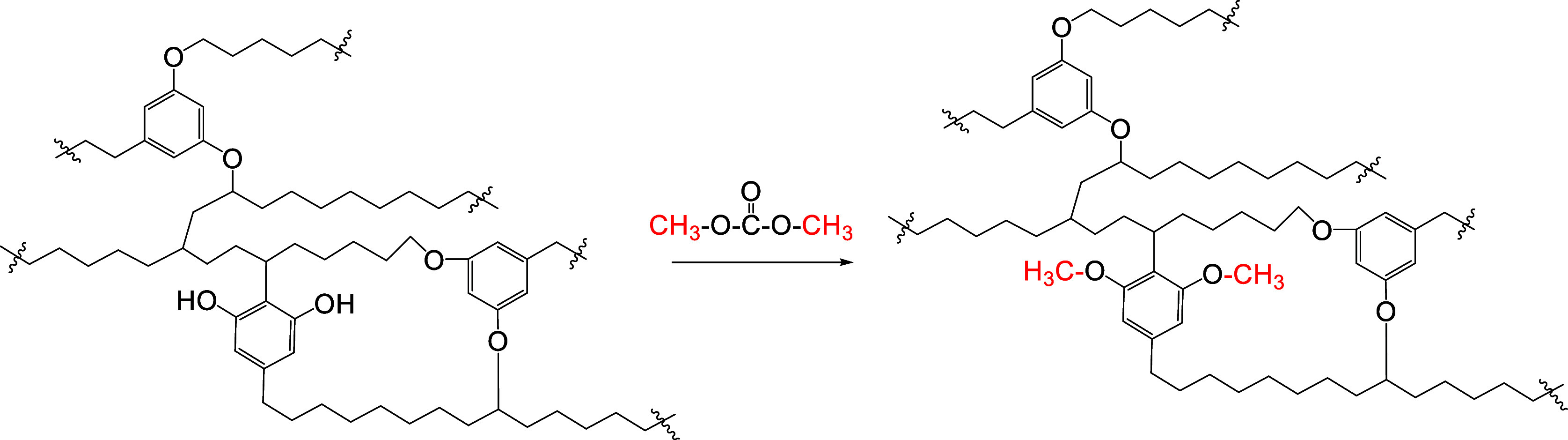
Methylation of Kukersite “Molecule” Fragment

The amount of free resorcinolic OH groups is also an important factor in determining the chemical properties of kukersite, as these groups make the kerogen structure sensitive to oxidation. Methylation of the phenolic OH groups can be used as a protective strategy and may allow the resorcinolic rings to survive various chemical transformations, especially oxidation.^86,87^ The alkylation of kerogen may also be used before the thermal processing of oil shale: introducing long-chain alkyl groups onto the hydroxyl sites of the kerogen may result in the generation of novel chemical compounds upon the thermal decomposition process. The obtained oil, which is rich in long-chain resorcinolic ethers, may have good characteristics for diesel oil after purification. These possibilities need further investigation.

An *O*-acylation of kukersite with acetic anhydride in pyridine was also carried out by Aarna and Lippmaa to determine the number of free OH- groups in kerogen.^37^ They found that the number of free OH groups is 0.22 equivalents in 100 g of kerogen. This means that there are 12.6 free OH groups per one “molecule” of kukersite. That is slightly less than expected based on the Lille-Blokker model, which predicts 14 free phenolic OH groups. An important conclusion from these data is that nearly 45% of all resorcinolic oxygen atoms appear as free OH groups.

Acylation may also change the properties of kukersite kerogen, so it can be used for the protection of the phenolic OH-groups in mild oxidation. Easily removable acyl groups offer additional opportunities for further chemical transformations of kerogen.

### 2.5 Destruction of Phenolic *O*-Ether Bonds

The resorcinol phenolic ethers present in native kukersite kerogen can potentially be dealkylated through chemical means. When studying the kukersite structure, Raudsepp conducted comprehensive work on the cleavage of ether bonds in kerogen.^36^ He found that hydrogen iodide cleaves ether bonds in kerogen at elevated temperatures and disrupts aromatic rings at higher temperatures. He found that 53 mmol of HI was consumed for 1 g of kerogen. He did not draw any conclusions from his results, and it was later stated by Fomina et al. that HI is too multireactive a reagent to determine quantitatively phenol ethers and aromatic rings.^88^

Aarna and Lippmaa treated kukersite kerogen with AlBr_3_ under nitrogen to split the phenolic ether groups in ampoules at 100 °C for 4.5 h. They found that 0.36 equivalents of the oxygen atoms in kerogen appeared as phenolic ether linkages and the ratio of substituted to free phenolic OH groups was around 0.6, slightly lower than the value estimated for free OH-groups.^37^ It is also important to notice, that the cleavage of the kerogen-phenol ether bond by treatment with AlBr_3_ is accompanied by partial degradation of the entire polymeric structure of the kerogen. Therefore, the number of ether groups may be slightly overestimated. This was evidenced by the formation of an ether-soluble substance accounting for more than 80% of the initial material during the course of that degradation, as well as gases, with a predominance of C_n_C_2n+2_.

As a side note, the most common reagent for cleaving aromatic ethers, BBr_3_, was not used in these studies. Nevertheless, ether bond cleavage could be a very valuable option for solubilizing kerogen for further transformations. This approach may be especially useful prior to oxidation, potentially leading to higher yields and different product compositions. All these possibilities require further investigation.

### 2.6 Nitration

The aromatic subunits of kerogen may undergo nitration via electrophilic substitution reactions. In addition, the hydroxyl groups can form nitric acid esters. Paul Kogermaǹs work also included experiments on the nitration of kerogen.^89,90^ The nitration of kukersite kerogen with 57% nitric acid at 100 °C for 1 h resulted in the formation of an acetone-soluble fraction amounting to 18% of the kerogen amount. When the reaction was carried out under the same conditions but together with sulfuric acid addition to the nitration mixture, then the acetone-soluble fraction increased to 44.3%. It is very likely that in first case ester formation predominates while in the second case nitration of the resorcinol ring together with some destruction of the kerogen backbone occurs.

The nitration of kukersite kerogen was investigated in more detail by Fomina, et al.^88^ They used different nitrating mixtures at a temperature of 20 °C and observed a 24% increase in the kerogen mass with a nitrogen content around 6%. At higher temperatures the oxidative destruction of kerogen dominated, leading to dicarboxylic acids. These experiments show that the nitration of kerogen is possible. No information on the nitrated products and properties of the nitrated kerogen is available. It can readily be suggested that nitration of kerogen should be carried out with special caution and under strict safety measures. So, it is difficult to propose practical uses for that process.

### 2.7 Oxidation

Oxidation of kukersite kerogen may destruct functional groups that are labile to oxidation (such as double bonds, aliphatic hydroxyl groups, etc.) as well as the unique resorcinolic units of kukersite kerogen. Oxidation is the most studied approach for kukersite direct chemical transformation. In order to elucidate the structure of kukersite, Kogerman et al. oxidized kukersite kerogen with alkaline potassium permanganate at 30 °C and observed a slow reaction with the formation of CO_2_ (35.6%), oxalic acid (43.8%) and acetic and other carboxylic and dicarboxylic acids (21.6%). However, he did not observe the formation of benzene-carboxylic acids. From this, he concluded that alkyl benzenes likely do not occur in the structure of kukersite kerogen.^81,90^ That conclusion fits well with the Lille-Blokker model.

Fomina et al. oxidized kukersite with potassium permanganate and elucidated the formation of mono-, di- and tricarboxylic acids in the process.^91–93^ Using a two-stage oxidation sequence, they were able to transform 38.4% of the kerogen carbon to a mixture of dicarboxylic acids.^88^

Several years later Bajc et al. also carried out a stepwise oxidation of kukersite kerogen with KMnO_4_ under very mild conditions in 33 steps and observed the formation of formic-, acetic-, di-, tri- and tetra-carboxylic acids.^94^

Proskurjakov et al. studied the air oxidation of kukersite kerogen under an air pressure of 40 bar at temperatures of 150–175 °C in water and observed the formation of C4–C10 dicarboxylic acids in yields of 4–12% from the initial kerogen^57^ (recalculated by Kaldas et al.^13^). We also studied the wet air oxidation of kerogen and obtained similar results.^95–97^ The noticeably lower yields of organic acids obtained were attributed to the overoxidation of the soluble product, which is unavoidable under the given conditions due to the radical nature of the process.

Blokker et al. studied the oxidation of kerogen with RuO_2_ and proposed that oxidation-labile resorcinol subunits oxidize to acetic acid, formic acid and CO_2_, leading to the depolymerization of the cross-linked native structure of kerogen. The primary oxidized material oxidizes further to carboxylic acids, dicarboxylic acids etc., and CO_2_/H_2_O. In Scheme 8 the oxidation of a simplified kerogen unit is presented (modified from ref 52). It was pointed out that during such oxidative destruction of the kukersite backbone, the carbon at which the alkyl chain is attached to the resorcinolic ring remains with the molecule of the dicarboxylic acids.

**Scheme 8:**
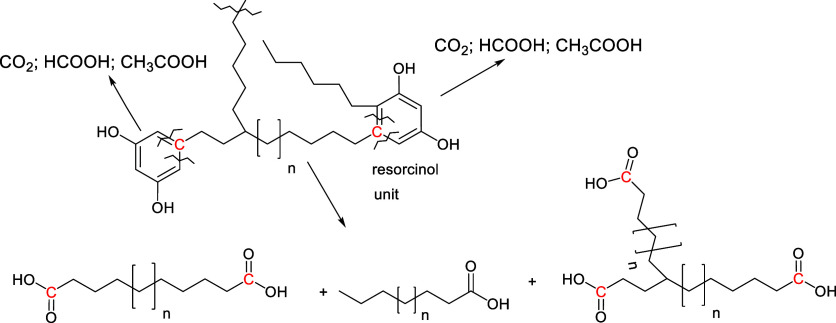
Oxidative Destruction of Kukersite Kerogen

Building on earlier work, Fomina et al. carried on in-depth studies using nitric acid, this time focusing on the oxidation of kerogen.^58,98^ It was found that, using 99% HNO_3_, dicarboxylic acids were formed in up to 55% yields. However, this approach was soon acknowledged as unreasonable due to technological risks and considerable nitration of kerogen and oxidation products in the course of oxidation. They carried on with the oxidation experiments of kukersite with 57% HNO_3_ at 90–95 °C for 4 h, which afforded dicarboxylic acids with a 17.6% yield per kerogen. At a temperature of 130 °C and a pressure of 50 bar, the yield of the dicarboxylic acids mixture increased up to 43.5%.^58,88^ Moreover, the separation and purification of the obtained dicarboxylic acids have also been described.^99,100^

We developed a technology of oxidation of CaCO_3_-free kukersite concentrate with diluted HNO_3_ in a continuous flow reactor with steady yields of dicarboxylic acids of around 25% from kukersite kerogen.^101^ Demonstrating that the oxidative production of dicarboxylic acids from complex solid materials like kerogen, using nitric acid, is adaptable to modern chemical industry, being both fast and scalable.

By compiling the results from different oxidation methods, it becomes evident that the relative ratios of the formed individual dicarboxylic acids in the crude oxidation mixture vary considerably (Figure 1). KMnO_4_, which provides the highest yields, results in nearly equal amounts of dicarboxylic acids with hydrocarbon chain lengths ranging from four to seven (C4 – C7). Oxidation with 57% HNO_3_ affords the dicarboxylic acids C5, C6, C7 and C8 in almost equal amounts of 20% each. During oxidation with air (WAO) radical processes dominate, leading to the rapid degradation of already-formed dicarboxylic acids.^102^ As a result, the product is predominantly composed of low-molecular-weight acids, such as C_4_ and C_5_. In general, it was observed that at lower temperatures the amount of shorter C4 and C5 dicarboxylic acids decreases, while at higher temperatures and harsh conditions the relative amounts of these dicarboxylic acids increase.

**Figure 1:**
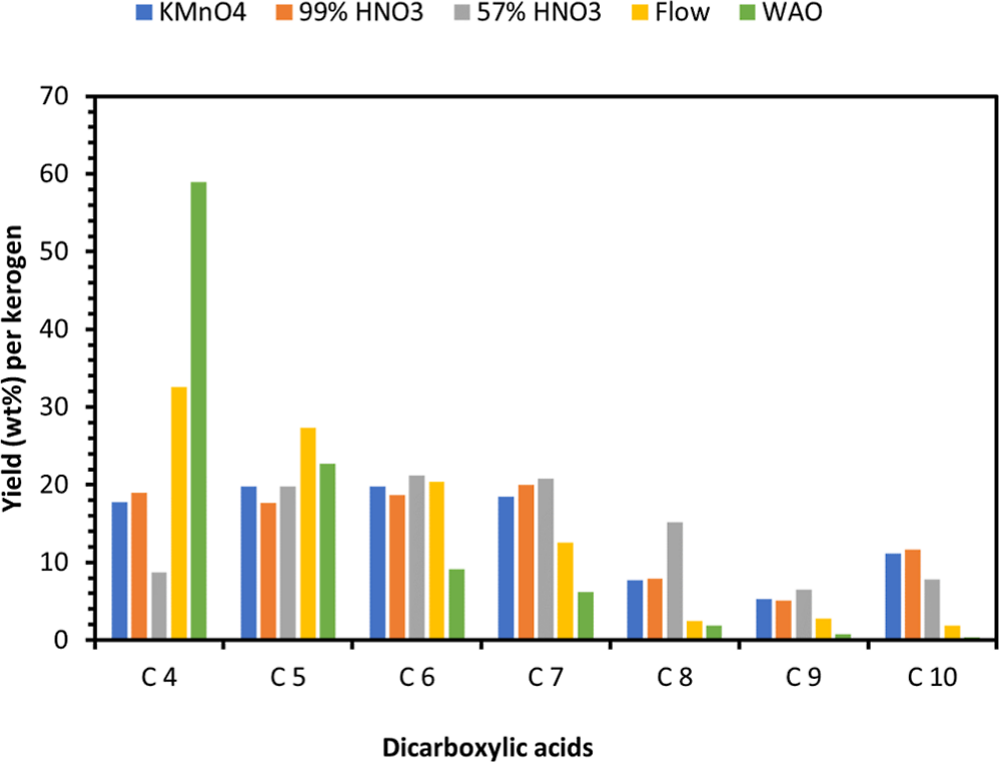
Relative dicarboxylic acid ratio from the oxidation of kukersite with different oxidizers.

All reviewed publications also reported that, compared to aliphatic dicarboxylic acids, the amount of aromatic dicarboxylic acids formed during oxidation was very low or undetectable. This suggests that aromatic structures are extensively degraded in oxidative conditions, regardless of the oxidizing agent used. Therefore, future studies might consider the idea of derivatizing (ex. methylating) the native kerogen prior to oxidation, which may afford new possibilities for the selective oxidation of kukersite kerogen. In the Lille-Blokker kerogen model, half of the resorcinol OH groups are free and undergo oxidative destruction of the resorcinolic unit (Scheme 1). If the phenolic OH group in the basic resorcinol subunit is alkylated, the protected resorcinols should tolerate mild oxidation and may undergo benzylic oxidation in the side chain instead of degradation according to Scheme 9.

**Scheme 9:**
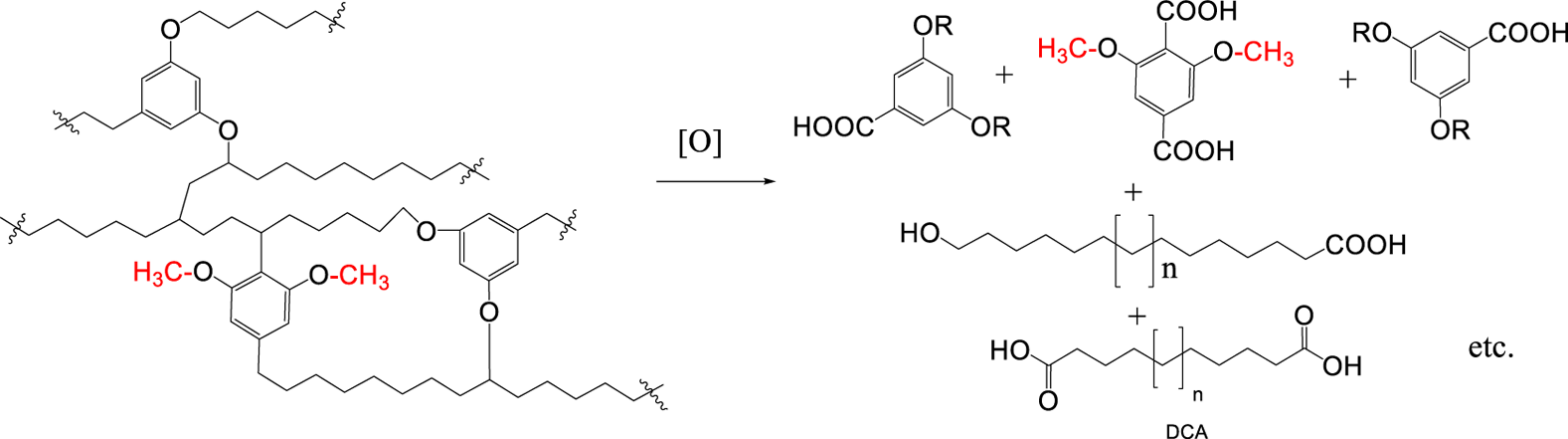
Possible Transformation Pathway of Methylated Kukersite

That approach may give us an opportunity to develop a CO_2_-emission-free process of the oxidative destruction of kukersite and to obtain both aromatic carboxylic acids and aliphatic di- and tricarboxylic acids.

### 2.8 Sulfonation

The sulfonation of kukersite kerogen affects its hydroxyl groups and may also sulfonate the aromatic rings via electrophilic sulfonation. No substantial research has been published on the sulfonation of kerogen, except the work by Cheshko et al.^103^ It was reported, that the treating kukersite kerogen with 95% H_2_SO_4_ afforded a sulfonation reaction where the amount of sulfonated kerogen reached its maximum at a reaction temperature of ∼120° (4 equivalents/g, Scheme 10). Furthermore, the sulfonated kerogen obtained was reported to exhibit ion-exchanging properties and could potentially be used as an ion exchanger.

**Scheme 10:**
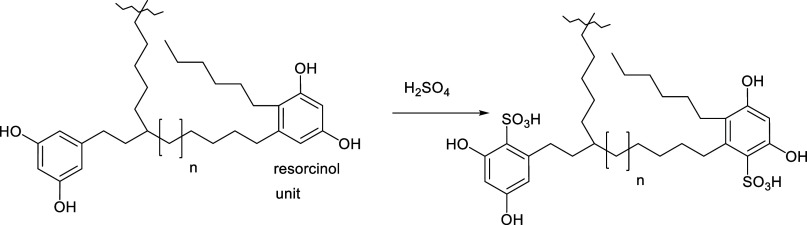
Sulfonation of a Simplified Kukersite Basic Subunit

### 2.9 Chloromethylation and Hydroxymethylation

Chloromethylation and hydroxymethylation reactions affect the aromatic subunits of kerogen through electrophilic substitution reactions. Aarna and Lippmaa used a chloromethylation reaction on kukersite to evaluate the number of aromatic rings in kerogen, based on the observed increase in sample mass. They reported that at least 15% of the carbon in kerogen is present in aromatic rings. Subsequent experiments with different reagents led them to revise their initial estimate, concluding that around ∼19% of the carbon atoms are aromatic, which is very close to the Lille-Blokker model.^37^

Chloromethylation is a very valuable tool for the modification of kerogen: the formation of chemically reactive benzyl chlorides intermediates opens up pathways for various chemical transformations to derive value-added materials with desired properties directly from kukersite kerogen (Scheme 11).

**Scheme 11:**
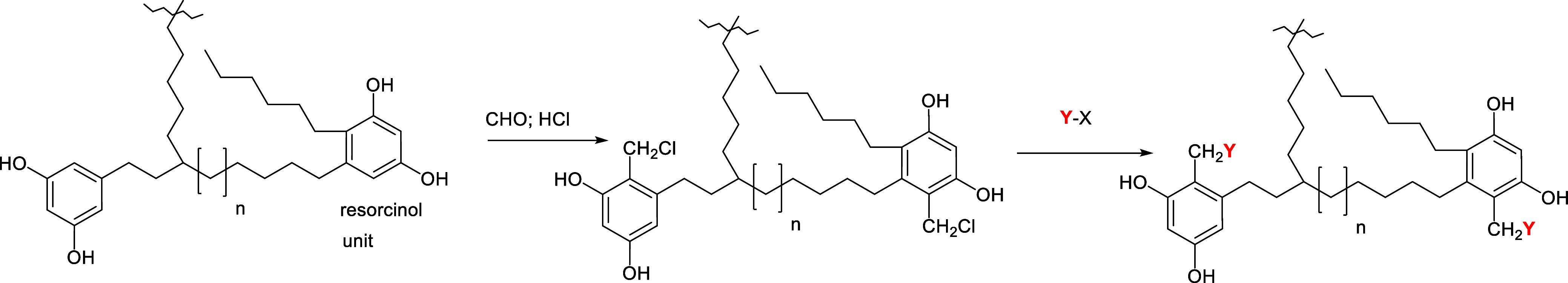
Chloromethylation of a Simplified Kukersite Basic Subunit

A parallel can be drawn with the hydroxymethylation reaction, which is often used in lignin chemistry^70,71^ to introduce additional OH groups into its complex polymeric structure. As a result, mainly the reactivity^104,105^ but also other properties of the polymer are altered, opening up new opportunities for chemical transformation. However, this reaction has not yet been applied to kukersite.

## 3 Conclusion

Traditional uses of kukersite—burning for energy and thermal decomposition for oil—are not the only possible ways to utilize this abundant and readily available organic raw material. Derivatization of its native chemical structure, followed by other targeted chemical transformations, opens new opportunities to obtain various chemicals and new materials from kukersite kerogen in more economical and environmentally friendly ways.

The main idea—to valorize the valuable subunits of native kukersite—has enormous potential. The positive and encouraging results of oxidative destruction of kukersite, as demonstrated by the successfully developed oxidation technology, represent just the first example proving the value of this approach. However, this method also needs improvement to preserve the valuable resorcinolic units and to reduce CO_2_ emissions from the process.

As shown above, halogenation, alkylation and acylation, chloromethylation, and sulfonation may also become useful tools to convert kukersite into valuable compounds and materials. It is therefore evident that many chemical transformation methods successfully applied to lignin may also yield interesting outcomes when used with kukersite kerogen. The examples of lignin conversion can serve as a guiding force for new studies in this direction.

Additional research and larger scale experiments are needed to develop new materials, chemicals, and corresponding technologies for utilizing kerogen as an alternative resource. After that their actual competitiveness can be properly assessed.
